# Evaluation of Occupational Health Risk Management and Performance in China: A Case Study of Gas Station Workers

**DOI:** 10.3390/ijerph19073762

**Published:** 2022-03-22

**Authors:** Muhammad Mohsin, Hengbin Yin, Weilun Huang, Shijun Zhang, Luyao Zhang, Ana Mehak

**Affiliations:** 1 College of International Finance and Trade, Zhejiang YueXiu University of Foreign Languages, Shaoxing 312000, China; mohsingrw@hotmail.com; 2 School of Finance and Trade, Wenzhou Business College, Wenzhou 325035, China; huangwl@wzbc.edu.cn (W.H.); 00204333@wzbc.edu.cn (S.Z.); 3 School of Business, Hanyang University, Seoul 04763, Korea; zhangluyao@hanyang.ac.kr; 4 College of Fisheries, Ocean University of China, Qingdao 266003, China; ana.mehak@yahoo.com

**Keywords:** occupational health, health risk assessment, occupational accidents, risk management, IPA, AHP

## Abstract

China has a large number of gas stations, with which thousands of workers are associated. There is abundant online literature documenting the various occupational health risks these workers face. However, this literature has many flaws to address, and it falls short of suggesting measures to manage these risks. This study strives to fill that gap, and aims to improve the occupational health of gas station workers through comprehensive risk management and performance analysis. To this end, a reasonable volume of reliable data, i.e., 208 completed questionnaires, were analyzed through current statistical routines, viz., fuzzy Analytical Hierarchy Process (AHP) and Importance Performance Analysis (IPA). These methods were employed to hierarchically organize the main factors and sub-factors of physical risk management, chemical risk management, biological risk management, physiological risk management and psychological risk management according to their appraised importance, and screen out the risk management stratagem for priority improvement. Research findings reveal that chemical risk and biological risk response schemes have the lowest performance, and need to be prioritized for improvement. Furthermore, this study argues that we can safeguard the occupational health of gas station workers through appropriate risk management strategies. It also elaborates on implications, limitations and future research directions.

## 1. Introduction

A plethora of existing online literature documents the diverse kinds of occupational health risks faced by gas station workers. For instance, Lim et al. [1] analyzed emission situation of volatile compounds. Cezar-Vaz et al. [2] found that prolonged exposure to benzene (lower series, i.e., BTEX) can increase chances of deoxyribonucleic acid damage of the lymphatic system. They reported higher concentrations of ethyl benzene, toluene, and benzene in the exposed group as compared to the control group. Likewise, studies indicate that gas station workers often suffer from noise-related health problems, including tinnitus, dizziness, and decreased hearing acuity [3]. Work overload destroys workers’ environment-coping competencies, producing emotional and stress-related changes such as anxiety, irritability, sadness and depression [4]. Besides, Mao-kui and Hong-jun [5] proposed various approaches to encounter fire at gas stations. The term “risk” generally symbolizes diverse factors having the potential to affect a worker’s quality of life [2,6]. Workers have acute feelings about risk factors through subjective judgment, a phenomenon called risk perception [7]. Risk perception is a rational outcome of workers’ interaction with their ambient environment [8]. This feeling relies on psychological evaluation of work-related environmental factors [2].

Risk perception consists of two fundamental features, i.e., the extent of possible loss and the chance of its incidence [8]. Because of these characteristics, different people have dissimilar perceptions about risk factors. That’s why people feel an analogous risk in different circumstances, and the same person can perceive risk quite differently in diverse conditions [9]. Risk perception aptitudes increase safety levels [10]. Unsafe or irresponsible behavior is the reported cause of occupational accidents [11]. Poor risk perception is a contributing factor to increasing occupational accidents [12] that affect society, health and economy both in developed and developing countries [13]. Understanding occupational risks is of prime importance to prevent such accidents [7], and exploring risk factors and management measures that have an impact on workers’ health is indispensable to improving their work quality. By doing this, we can diminish or even obliterate risk factors confronted by workers in their work environment, and help enhance their occupational health levels.

Occupational accidents can happen at gas stations due to various risk factors [14] such as physical risks, chemical risks, biological risks, physiological risks and psychological risks. Several health issues related to noise can be observed at gas stations. These problems can result in irritability, reduced hearing and physical anxiety [14]. Health risks arising from chemical exposure at gas stations are well documented in the literature. For instance, respiratory complications can occur because of biological agents present at gas stations [15]. Monotonous movements, poor posture and prolonged working hours can hurt the limbs (upper and lower) and cervical spine. In addition, intoxication of digestive tract and airway can occur due to benzene. Likewise, formation of skin lesions is associated with the prevalence of benzene at gas stations [16,17,18]. Workers are exposed to the risk of blood cancer, i.e., leukemia (lymphoblastic and myeloblastic) and cancer of the lymphatic system, i.e., non-Hodgkin lymphoma [19,20]. Reported types of risks are elaborated in the coming sections.

In China, the gasoline industry contributes significantly to the national economy [21]. This country has a large number of gas stations, with which thousands of workers are associated [22]. Several researchers have conducted studies on the gasoline industry in China. However, most of their work either studies other aspects of this industry or does not comprehensively assess risks faced by gas station workers—describing risk management measures in a limited way. For instance, Xuhui [23] described various techniques to improve the safety of buried gas tanks by discussing tank structure, design, leakage and inspection measures. Similarly, Li and Pan [24] strived to improve the digital transformation of gas stations in China. They studied various factors affecting digital transformation, identified problems, and suggested possible solutions. Li et al. [25] reported contamination due to heavy metal dust particles at gas stations in Xi’an, China. Their findings indicated that Cr, Cu, Pb, Cr and Zn mainly originate from the traffic flow, and pose significant cancer risk to workers. Prior published literature related to the gasoline industry of China has many limitations. First, most of the documented literature is in Chinese, which limits access to an international audience. Second, most of this reported work does not address occupational health risks or management of gas station workers. Third, studies on gas station workers generally focus on a single or limited number of risk factors, without comprehensively describing ways to manage them. Studies on risks faced by gas station workers and their management are essential for improving occupational work conditions in China, the fastest-growing economy in the world.

Various statistical routines can be applied to data-describing risks. The choice of model and statistical procedure depends on applicability, reliability and nature of study. Multi-criteria models are the most dependable and trustworthy tools to rank and prioritize risks for their management. Among the various multi-criteria approaches available, the Analytical Hierarchy Process (AHP) and Importance Performance Analysis (IPA) are the most recognized and reliable risk management tools [26]. That’s why studies related to gas stations employ these methods frequently [27,28]. The AHP method was proposed by Saaty in 1977. In 1980 and 1988, this method was further revised and refined by him [29,30,31]. AHP enables managers to identify the most suitable option among various possible choices. It also helps in measuring performance consistency [32]. This method uses the pairwise comparison technique by using rational scores and weights, helping decision-makers arrive at a reliable risk management strategy. Published research works have employed the AHP method for several reasons. First, this method has the ability to evaluate data inconsistencies and propose instinctive appeal while being flexible in operation. Second, it suggests hierarchy, and efficiently helps decrease decision bias. Third, it makes a logical pairwise comparison. Fourth, this method is specially designed to study risks along with associated uncertainties [33,34]. Due to the above-mentioned advantages, we have employed AHP and IPA methods in this study too.

The core objective of this study is to improve the occupational health of gas station workers through risk management and performance analysis. It also provides a framework of actions to encounter these risks. According to the available empirical evidence, physical risks, chemicals risks, biological risks and physiological risks are the main risk factors perceived by gas station workers [2] in the work environment. The following section presents a comprehensive review of these risk factors.

## 2. Literature Review and Conceptual Paradigm

### 2.1. Risk Factors

According to prior empirical evidence, physical risks, chemicals risks, biological risks and physiological risks are the main work environment risk factors perceived by gas station employees [2].

#### 2.1.1. Physical Risks

Ergonomists argue that an unhealthy working environment, mainly generated by radiation, high noise and exhaust emission, affect the worker’s quality of life and organizational productivity [35]. At gasoline stations, station attendants, service recipients and car drivers are among those exposed to various occupational hazards [36]. Studies indicate that gas station workers very often experience noise-related health problems, including tinnitus, dizziness and decreased hearing acuity [3]. Vibrations emanating from equipment and traffic constitute a real risk to worker health [2]. As a potential safety and health hazard, heat stress has also been identified in the literature. Studies conclude that heat and noise have a combined effect on human functions [37,38]. At gas stations, the temperature is usually as high as 30.6 °C and the noise level goes up to 90 dBA. Sources of artificial light radiation include solar lamps and fluorescent lamps at the workplace, and these can cause skin cancer [39]. In this study, the physical risk factors to which gas station staff are exposed include radiation (ionizing radiation and non-ionizing radiation), noise from vehicles, extreme air temperatures (hot and cold) due to weather changes, and vibration.

#### 2.1.2. Chemical Risks

Available literature shows that acute or long-term exposure to chemical compounds at gas stations may lead to systemic health consequences, including haematological, respiratory, reproductive, immunological, dermatological, renal and central nervous system pathologies in humans [40]. Li et al. [25] assessed heavy metals contamination in dust at gas stations located in Xi’an, China. Their findings indicated that Cr, Cu, Pb, Cr and Zn mainly originate from traffic flow, and pose a significant lifetime cancer risk to workers. Gas station workers are frequently exposed to toxic petrochemical substances, including volatile organic compounds such as benzene, toluene, ethylbenzene, xylenes and methyl tertiary butyl ether (MTBE), mainly through dermal absorption, inhalation and contact with the eyes [41]. Empirical evidence validates that short-term exposure to chemical products (heavy metal, fuel, oil, etc.) is likely to cause skin irritation [42]. Biological agents can cause respiratory tract infections [15]. Gasoline vapor and mist are readily inhaled or ingested when released into the air during refining, gasoline transfer, and leaks from storage containers and loading equipment. Fumes and/or gas inhalation are the leading cause of sneezing, allergic rhinitis and asthma among gas station workers [1,43,44]. In addition, results of studies aiming to assess the prevalence of self-rated ill-health conditions among gas station workers have shown that eye complaints due to contact with substances such as dust, gasoline, grease and alcohol were somewhat higher in magnitude than official reports from many countries [45].

#### 2.1.3. Biological Risks

A health risk analysis of gas station works had previously shown that biological risks mainly include bacteria, viruses, parasites, fungi, bacilli and protozoa [2]. Many chemical agents that may cause bacterial and viral infections are present in the occupational environment. They cause many adverse effects to human health resulting in cancer, cardiovascular diseases, etc. [46]. Besides that, other factors can also aggravate biological risks such as physical, chemical and physiological stresses [47]. For instance, extreme temperature, noise and pressure changes may help bacteria, viruses and parasites to breed at gas stations with poor sanitary conditions [18,48].

#### 2.1.4. Physiological Risks

Studies demonstrate that improper posture and manual handling may lead to injury and pain of the cervical spine, and upper or lower limbs [2]. Repetitive movements and standing for a long time can easily cause trauma and musculoskeletal disorders, including back, shoulder and knee problems; repetitive strain injuries; stress; fatigue; and muscle strain [49]. Physiological stressors associated with slippery surfaces are also reported by gas station workers, most such complaints coming from refueling employees [47]. In addition, previous estimates also suggested that the physiological risk of collision between cars and gas station workers should not be ignored [2]. Thus, all these identified problems may bring physiological stress to gasoline station workers.

#### 2.1.5. Psychological Risks

Recent research findings indicate that working at a gas station is very likely to generate stress responses due to unbalance between psychological agents and worker skills [50]. Although attention has been given mainly to chemical and physical risks, fuel station workers are also exposed to real psychological risks. Increasing job demands and interaction with customers derived from ferocious market competition between fuel companies favor a rise in worker responsibilities. Besides, constant change in work conditions implies new workloads for workers [51]. Work overload destroys workers’ environment-coping competencies, which may produce emotional changes and symptoms of stress such as anxiety, irritability, sadness and depression, which is identified as a psychological and social risk factor [4]. More than that, chronic stress is likely to cause psychological stress and mental disorders [52]. Remuneration is one such psychosocial factor that leads to negative emotions in workers, mostly relating to dissatisfaction with the balance between expected work effort and the reward. Several studies have pointed out that the disparity between these job features is a psychosocial risk factor leading to other problems such as fatigue and burnout [51].

### 2.2. Risk Response Stratagem

Taking the five types of perceived risks above into consideration, intervention strategies should be formulated and implemented. Nowadays, several practical solutions to problems and corrective actions to specific risk agents have been deployed to improve gas station workplace conditions.

#### 2.2.1. Physical Risk Management

The employee’s operating environment temperature, the intensity of illumination, the concentration of harmful gases, working space and risk zoning should comply with relevant provisions of the state’s safety standards. Gas stations are frequently encouraged to promote human–machine system design to address environmental and public health issues [47]. Cognizant of the harmful effects of ionizing and non-ionizing radiation, strategies have been formulated to utilize personal protective devices (PPD) [2]. Besides PPD, several fuel stations have strengthened hardware configurations to minimize risks caused by noise-induced hearing loss and vibrations [53].

#### 2.2.2. Chemical Risk Management

Corporate organizations can limit their workers’ exposure to hazardous chemicals by establishing and implementing unambiguous occupational safety and health (OSH) policy statements to protect personnel against exposure [54]. It is vital to provide sufficient resources, including personnel training, personal protective equipment and medical supervision, to implement these plans effectively. The establishment of clear communication lines for emergency preparedness and hazard characterization is also important. Currently, levels of chemical products in workplace air are much lower than in the past because of the adoption of safety measures at gas stations. Due to the availability of respirators and other protective gadgets such as masks, gloves, aprons and boots, the number of workers at gas stations who may have symptoms of chemical poisoning and related health risks has decreased [6]. Besides that, safety glasses and goggles are used to protect the eyes from substances such as dust, detergent, grease, gasoline and alcohol [2]. With growing awareness on the importance of monitoring gasoline concentration at the workplace, proper ventilation systems and procedures for equipment maintenance have been developed to prevent joint leakage and reduce human exposure [55].

#### 2.2.3. Biological Risk Management

It is essential to provide equitable access to health facilities, services, and medical supervision to mitigate biological risks effectively. To cope with viral risk, studies emphasize healthy eating and improving the body’s natural defense [2]. Some gasoline stations implement medical surveillance at the workplace [6]. According to research findings, washing hands regularly should be encouraged for gas station staff [2]. Gas companied do efforts to increase their employee’s awareness about their ambient environment. Employees are encouraged to take adequate protective measures at workplace.

#### 2.2.4. Physiological Risk Management

Employees operating environment, operating space and risk zoning should conform with the safety standards. Studies associated with workers’ perceived risk demonstrated that managing materials scattered on slippery floors and working spaces is a good strategy for minimizing the risk of slick at fuel stations [2]. Besides, gas stations should try to alleviate the risk of collision between cars and workers by ensuring proper vehicles flow [56]. Proper work shifts and schedules should be established to eliminate severe fatigue problems caused due to repetitive movements, and long periods of standing required for the job [49]. Exercising regularly has also been proposed as a control measure to address poor posture issues and repetitive strain suffered by fuel station attendants [2,6]. In addition, since ergonomics has been used to reduce occupational injuries and musculoskeletal disorders, a new computer software package for ergonomic assessment procedure has been developed [53]. Practical application of ergonomics in the gas station work system design may balance worker safety and task demands.

#### 2.2.5. Psychological Risk Management

Previous findings suggest that workers’ psychological well-being is endangered by increasing occupational stress [57]. Abnormal pressure due to excessive work leads to emotional changes such as anxiety and depression, which justify the need for health prevention actions. These activities and programs include organizational time-outs, proper job design, specific job roles, assurance of job security, greater latitude, and support for employees [58]. For instance, ergonomics application in the work system design can ensure mental well-being and job satisfaction by balancing worker characteristics and task demands [59]. It is also imperative that gas station workers be encouraged to participate in activities such as workshops that help eliminate and reduce occupational stress [2].

## 3. Materials and Methods

### 3.1. Research Framework

**Figure 1:**
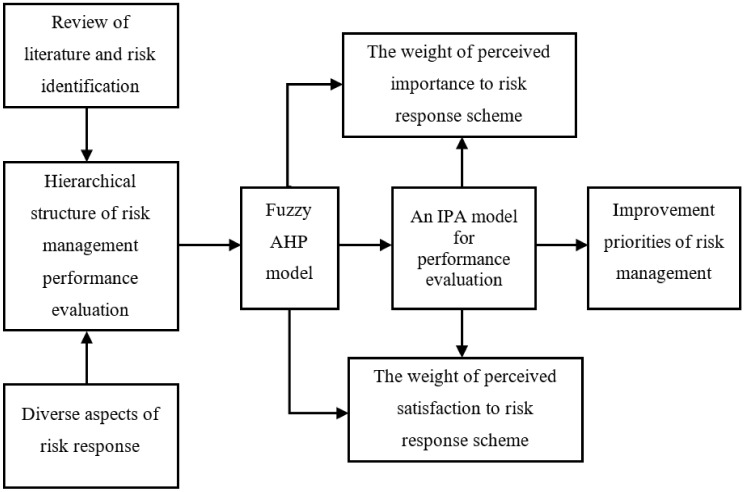
The research framework.

The available literature fails to comprehensively access, describe, compare and suggest practical measures to counter risks faced by gas station workers. Thus, the core objective of this study is to identify, rank and prioritize risk factors and safeguard the occupational health of workers. Figure 1 presents a basic research framework of this study.

**Figure 2:**
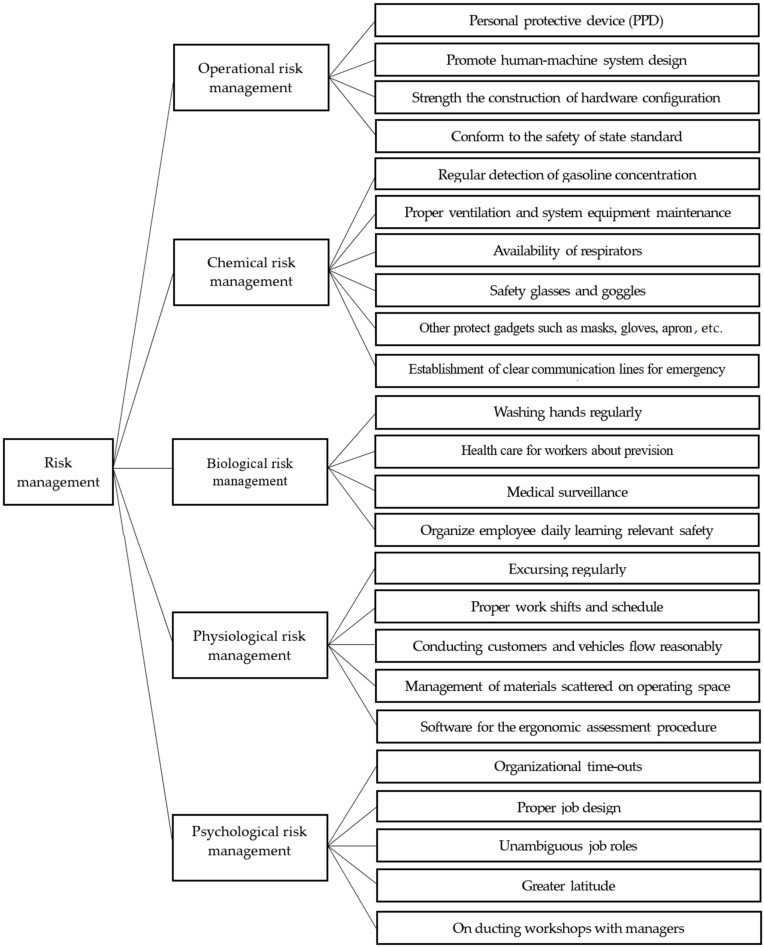
Two-layered hierarchy structure of risk management performance.

Considering the features of risk management and its performance, the corresponding hierarchy was constructed based upon relevant published literature. This hierarchy comprises two layers. The first layer consists of five risk management factors. The second layer consists of 24 risk management sub-factors (Figure 2). Later on, fuzzy AHP is applied to data for risk ranking. Subsequently, the IPA statistical method is used to prioritize risk factors for their effective management.

### 3.2. Questionnaire Design

A questionnaire based on a 9-point rating scale [30] was designed to fetch data for fuzzy AHP and IPA analysis. Depending on the hierarchical structure of risk factors and their sub-factors, a questionnaire comprising 5 criteria and 24 sub-criteria was drafted. In order to ensure reliability, readability and dependability of the questionnaire, it was reviewed by two professors working in this field of research. Later on, this questionnaire was vetted by three CEOs of gas stations to confirm that no important question was missed. The core objective of drafting this questionnaire was to identify, rank and prioritize the risk factors faced by gas station workers for better management. As many as 208 completed questionnaires suitable for analysis were collected from the Yangtze River delta region, i.e., Zhejiang, Shanghai and Jiangsu, through emails (142 with a 53% response rate), telephone calls (176 with a 79% response rate), and face-to-face interviews (113 with a 74% response rate) between 1 April and 31 August 2021.

### 3.3. Research Sample

Our goal was to collect a sufficient volume of data for this study. In total, we obtained 208 completed questionnaires suitable for statistical analysis. Details of the study subjects are presented in Table 1. As the research sample is central to this research, we have elaborated on it thoroughly. Besides, we received distinctive responses from workers during the data acquisition process. Therefore, we present our perceptions along with their possible reasons while explaining characteristics in the frequency analysis. When talking about marital status, workers mostly avoided saying separated. This is a local cultural response with reference to separation. However, this is of vital relevance to the study because lack of familial responsibilities allows single or separated workers to quickly quit the job if they find a better opportunity or face some problem. For this reason, we tried to fetch more data on married workers as they tend to work longer, and do more analytic thinking about their working conditions.

Among male and female workers, males mostly show more willingness to work at gas stations because of the nature of the work. Generally, gas stations are dominated by part-time workers who stick around for less than a year. Workers who continuously work, usually for more than two years, are promoted to managers. Managers have additional responsibilities compared to general workers, such as helping customers make membership cards and distributing coupons related to car wash and free engine oil change. We used the data collected from the Yangtze River delta region, i.e., Zhejiang Province, Jiangsu Province and Shanghai. This area is the most developed in China, and acts as a major socio-economic engine [60]. The dynamic economy of this region is of central importance to the Chinese government, and the gasoline industry of this region is very important and representative [61]. Therefore, we selected this region for the study. Additionally, workers work in two shifts, i.e., day and night, 12 hours per shift, at gas stations. Choice of selecting the time for working depends upon an available position as well as personal preference. Single people have a higher tendency to select the night shift as fewer cars visit gas stations during the night, allowing them more rest time. This study also finds that, middle school graduates usually work at gas stations to fill gas vehicles.

### 3.4. Fuzzy Theory and Analytic Hierarchy Processes (AHP)

The fuzzy AHP refers to a decision-making method that determines and applies different evaluation criteria for objective evaluation. As precise numbers or verbal expressions for evaluating measures can be ambiguous, inaccurate and subjective, fuzzy theory can be applied to supplement them [62]. It is a method that mathematically presents ambiguous and erroneous expressions in uncertain conditions or uncertain linguistic, numerical terms to enable explicit judgments [63]. Fuzzy theory is sometimes described as a set theory that introduces subjectivity to solve these problems. Several previous studies have employed AHP and IPA techniques to analyze risk management and risk performance at gas stations [64]. The main advantage of the AHP is its structured multi-attribute decision method that reduces bias in the decision-making process. As assessing scales are involved instead of measurements, fuzzy AHP can be used where there is a lack of measurement, such as modelling risk and uncertainty [65].

The first step of AHP is to identify risk factors and response schemes based on expert interviews and a review of the literature. Based on a review of the literature and interviews with experts, the second step is to determine the criteria for evaluating the effects of response schemes developed in the risk factors selected above. In the third step, this study used AHP to identify the relative weights of each criterion based on interviews with experts. Based on the results, the optimal candidate for gas station risk management was explored through the well-developed fuzzy method employed in this study [65].

### 3.5. The Weights of Risk Factors

In this study, for each hierarchical layer of risk factors and sub-factors, 208 pairwise comparisons were obtained. Most of the time, published literature employs the geometric or arithmetic mean to reflect the choices of multiple subjects. But these means rely on extreme values. On the other hand, in this study a fuzzy number is used to incorporate all the perceptions in a unified manner. In the first step, a geometric mean was estimated to denote opinions of study subjects [30,66]. Afterwards, in the second step, a fuzzy positive matrix was obtained by using the lowest measuring values and highest geometric mean [67]. Subsequently, considering this matrix, we employed fuzzy AHP approach to compute weights of risk factors.

#### 3.5.1. The Fuzzy Positive Reciprocal Matrix

We assumed $\tilde{A}={\left[{\tilde{a}}_{ij}\right]}_{n\times n}$ a fuzzy matrix, i.e., positive and reciprocal. In this equality ${\tilde{a}}_{ij}=\left[l_{ij},m_{ij},u_{ij}\right]$ represents $\left[l_{ij},m_{ij},u_{ij}\right]=\left\{\begin{array}{c} \left[1,1,1,\right],\;if\;i=j;\\ \left[\frac{1}{u_{ij}},\frac{1}{m_{ij}},\frac{1}{l_{ij}}\right],\;if\;i\neq j. \end{array}.\right.$

More plausibly, pairwise comparison can be denoted *n* Risk Factors when the subjects are *k*th. Employing this integrated method, fuzzy positive reciprocal matrix can be obtained by using all the 208 pairwise comparisons, i.e., *A^(k)^*, *k* = 1, 2, ……, 208. This matrix can be expressed as $\tilde{A}={\left[{\tilde{a}}_{ij}\right]}_{n\times n}$. In this equation, ${\tilde{a}}_{ij}=\left[\underset{1\ll k\ll 30}{\min}\left\{a_{ij}^{\left(k\right)}\right\},\;{\left(\prod_{k=1}^{30}a_{ij}^{\left(k\right)}\right)}^{1/30},\underset{1\ll k\ll 30}{\max}\left\{a_{ij}^{\left(k\right)}\right\}\right]$ stands for triangular fuzzy number. Moreover, *i* and *j* each of them represent values from 1 to *n*. By applying mathematical calculations, we can denote the fuzzy positive reciprocal matrix as follows [68]: ${\tilde{a}}_{ij}=\left\{\begin{array}{c} \left[1,1,1,\right],\;if\;i=j;\\ {\left({\tilde{a}}_{ij}\right)}^{-1},\;if\;i\neq j. \end{array}.\right.$

#### 3.5.2. The Local Weights of Risk Factors

In our study, we adopted a special method for determining local weights of risk factors. This method was proposed by Saaty [30], and uses the geometric mean normalization of rows. According to the description of Kaufinami and Gupta [68], we employed the following equality to get the geometric mean of triangular fuzzy numbers:

(1) $${\tilde{w}}_{i}={\left(\prod_{j=1}^{n}{\tilde{a}}_{ij}\right)}^{1/n}=\left[{\left(\prod_{j=1}^{n}l_{ij}\right)}^{1/n},{\left(\prod_{j=1}^{n}m_{ij}\right)}^{1/n},{\left(\prod_{j=1}^{n}u_{ij}\right)}^{1/n}\right],i=1,2,\ldots ,n.$$

where *i*th for risk factors varies from 1 to *n*. By using above equation, we can have $\sum_{i=1}^{n}{\tilde{w}}_{i}=\left[\sum_{i=1}^{n}{\left(\prod_{j=1}^{n}l_{ij}\right)}^{1/n},\sum_{i=1}^{n}{\left(\prod_{j=1}^{n}m_{ij}\right)}^{1/n},\sum_{i=1}^{n}{\left(\prod_{j=1}^{n}u_{ij}\right)}^{1/n}\right]$. Hence, fuzzy weight of *i*th for risk factors can be estimated as follows:

(2) $${\tilde{W}}_{i}=\frac{{\tilde{w}}_{i}}{\sum_{i=1}^{n}{\tilde{w}}_{i}}=\left[\frac{{\left(\prod_{j=1}^{n}l_{ij}\right)}^{\frac{1}{n}}}{\sum_{i=1}^{n}{\left(\prod_{j=1}^{n}u_{ij}\right)}^{\frac{1}{n}}},\frac{{\left(\prod_{j=1}^{n}m_{ij}\right)}^{\frac{1}{n}}}{\sum_{i=1}^{n}{\left(\prod_{j=1}^{n}m_{ij}\right)}^{\frac{1}{n}}},\frac{{\left(\prod_{j=1}^{n}u_{ij}\right)}^{\frac{1}{n}}}{\sum_{i=1}^{n}{\left(\prod_{j=1}^{n}l_{ij}\right)}^{\frac{1}{n}}}\right],\;i=1,2,\ldots ,n.$$

#### 3.5.3. Defuzziness Process

Calculated weight, i.e., *W_i_* is fuzzy of *i*th (*i* = 1, 2, …, *n*) for risk factors. So, first, we have defuzzied *W_i_* to *W_i_* (crisp number) by using the index proposed by Yager [69], $W_{i}=\left[l_{i}^{W},m_{i}^{W},u_{i}^{W}\right]=\left[\frac{{\left(\prod_{j=1}^{n}l_{ij}\right)}^{\frac{1}{n}}}{\sum_{i=1}^{n}{\left(\prod_{j=1}^{n}u_{ij}\right)}^{\frac{1}{n}}},\frac{{\left(\prod_{j=1}^{n}m_{ij}\right)}^{\frac{1}{n}}}{\sum_{i=1}^{n}{\left(\prod_{j=1}^{n}m_{ij}\right)}^{\frac{1}{n}}},\frac{{\left(\prod_{j=1}^{n}u_{ij}\right)}^{\frac{1}{n}}}{\sum_{i=1}^{n}{\left(\prod_{j=1}^{n}l_{ij}\right)}^{\frac{1}{n}}}\right],\;i=1,2,\ldots ,n$. The value of *i* varies between 1 and *n*. This index can also be denoted as $W_{i}=\left(l_{i}^{W}+2m_{i}^{W}+u_{i}^{W}\right)/4,i=1,2,\ldots ,n.$ Later on, we normalized *W_i_*, where 1 = 1, 2, …, *n*, as follows: $\;W_{i}=W_{i}/\sum_{i=1}^{n}W_{i},i=1,2,\ldots ,n.$

### 3.6. Performance Evaluation and Importance-Performance Analysis (IPA)

**Figure 3:**
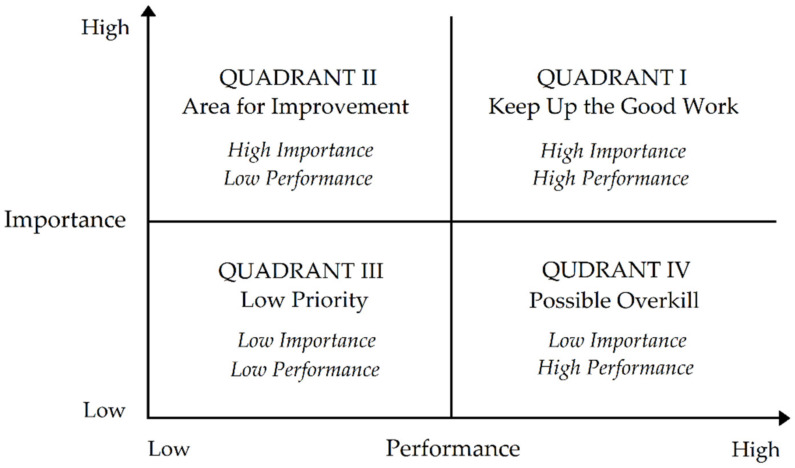
IPA model.

In order to evaluate the risk management performance, an IPA model was employed (Figure 3). This statistical routine is a popular matrix assertively used to recognize and rate risk factors depending on their importance by highlighting their comparative impact on corporate performance.

By using this grid, an organization can obtain perceptivity about the risk factors deserving improvement against those that have consumed plenty of resources but have a negligible impact on the overall organizational performance. Basically, this matrix plots “performance” and “importance” in a two-dimensional matrix consisting of four quadrants by plotting the former attribute on the *x*-axis and the latter one on the *y*-axis. Quadrant I denotes high importance as well as high performance. Thus, it complements competitive advantage, and is written off as “Keep Up the Good Work”. Quadrant II connotes high importance but low performance. It needs improvement through prompt attention, and is thereby termed as an “Area for Improvement”. Quadrant III, indicating squat ranks of importance and performance, designates the attributes that should be strategically “Low Priority”. Quadrant IV characterizes attributes that have low importance but high performance, and are thereby regarded as “Possible Overkill”. It means that resources employed by these attributes should be more rationally deployed in other quadrants (except Quadrant III).

## 4. Results

### 4.1. General Characteristics of Research Subjects

In respect to the general characteristics of research subjects, for marital status, 64 individuals (30.8%) were ‘single’ and 144 (69.2%) were ‘married’; for gender, 144 respondents (69.2%) were ‘male’ and 64 (30.8%) were ‘female.’ Regarding work experience, 80 individuals (38.5%) answered ‘1~3 years’, and 128 (61.5%) chose ‘over 3 years’. In terms of region, 96 participants (46.2%) were from ‘Zhejiang Province’, 56 (26.9%) were from ‘Shanghai’, and 56 (26.9%) were from ‘Jiangsu Province’. In terms of work shifts and schedule, 168 respondents (80.8%) chose ‘daytime’, while 40 (19.2%) answered ‘nighttime’. In regard to schooling, 16 individuals (7.7%) responded ‘middle school’, 56 (26.9%) chose ‘high school’, and 136 (65.4%) answered ‘higher education, incomplete’.

### 4.2. The Relative Importance and Priority Ranking of Main Factors

The relative importance and priority ranking of main factors followed the following order: ‘chemical risk management’ (0.341), ‘physical risk management’ (0.219), ‘psychological risk management’ (0.186), ‘biological risk management’ (0.137), and ‘physiological risk management’ (0.116). The consistency ratio (CR) of each factor was smaller than 0.1, which indicated consistency (Table 2).

### 4.3. The Relative Importance and Priority Ranking of Sub-Factors

**Figure 4:**
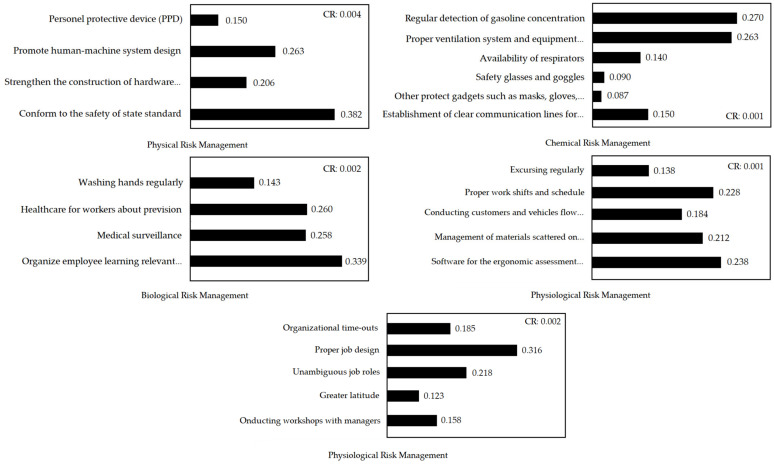
The Relative Importance and Priority Ranking of Sub-Factors.

Figure 4 portrays the relative importance and priority ranking of physical risk management sub-factors, which are in the following order: ‘conform to the safety of state standard’ (0.382), ‘promote human–machine system design’ (0.263), ‘strengthen the construction of hardware configuration’ (0.206), and ‘personal protective devices (PPD)’ (0.150). A CR value less than 0.1 indicated consistency. The sub-factors of chemical risk management displayed relative importance and priority, ranking in the following order: ‘regular detection of gasoline concentration’ (0.270), ‘proper ventilation system and equipment maintenance’ (0.263), ‘establishment of clear communication lines for emergency preparedness’ (0.150), ‘availability of respirators’ (0.140), ‘safety glasses and goggles’ (0.090), and ‘other protect gadgets, such as masks, gloves, apron and boots’ (0.087). The relative importance and priority ranking of sub-factors of biological risk management were in the following order: ‘organizing daily employee learning regarding relevant safety knowledge’ (0.339), ‘healthcare for workers about prevision’ (0.260), ‘medical surveillance’ (0.258), and ‘washing hands regularly’ (0.143). The CR value was less than 0.1, demonstrating consistency. The relative importance and priority ranking sub-factors of physiological risk management followed the order of ‘software for ergonomic assessment procedure’ (0.238), ‘proper work shifts and schedule’ (0.228), ‘management of materials scattered on operating space’ (0.212), ‘conducting customers and vehicle flow reasonably’ (0.184), and ‘excursing regularly’ (0.138). CR value less than 0.1 indicated consistency. The relative importance and priority ranking sub-factors of psychological risk management were in the following order: ‘proper job design’ (0.316), ‘unambiguous job roles’ (0.218), ‘organizational time-outs’ (0.185), ‘conducting workshops with managers’ (0.158), and ‘greater latitude’ (0.123). A CR value less than 0.1 demonstrated consistency.

### 4.4. Performance Analysis of Main Factors

According to the analysis, the performance of the main factors followed the order of ‘chemical risk management’ (0.341), ‘biological risk management’ (0.137), ‘physiological risk management’ (0.116), ‘physical risk management’ (0.219), and ‘psychological risk management’ (0.186) (Table 3).

### 4.5. Performance Analysis of Sub-Factors

**Figure 5:**
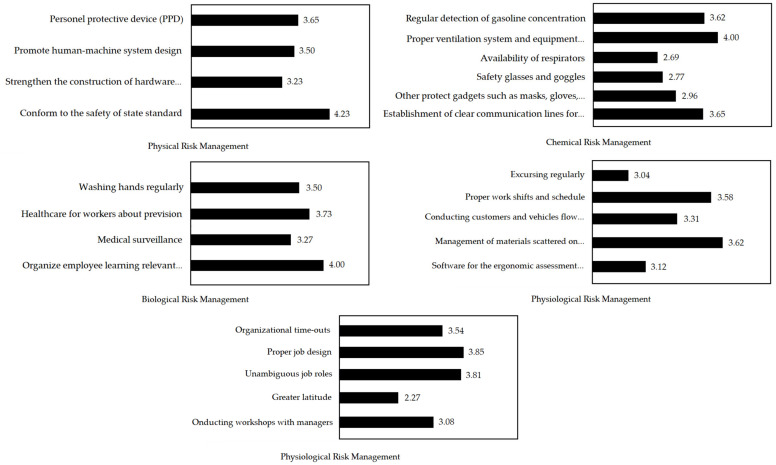
Performance Analysis of Sub-Factors.

The performance of sub-factors of physical risk management was in the order of ‘conforming to the safety of the state standards’ (4.23), ‘personal protective devices (PPD)’ (3.65), ‘promoting human–machine system design’ (3.50), and ‘strengthening the construction of hardware configuration’ (3.23). The performance of sub-factors of chemical risk management was in the following order: ‘proper ventilation system and equipment maintenance’ (4.00), ‘establishment of clear communication lines for emergency preparedness’ (3.65), ‘regular detection of gasoline concentration’ (3.62), ‘other protective wear, such as masks, gloves, apron and boots’ (2.96), ‘safety glasses and goggles’ (2.77), and the ‘availability of respirators’ (2.69). The performance of sub-factors of biological risk management was in this order: ‘organizing daily employee learning regarding relevant safety knowledge’ (4.00), ‘healthcare for workers about prevision’ (3.73), ‘washing hands regularly’ (3.50), and ‘medical surveillance’ (3.27). The performance of sub-factors of physiological risk management was in the following order: ‘management of materials scattered on operating space’ (3.62), ‘proper work shifts and schedule’ (3.58), ‘conducting customer and vehicle flow in a reasonable manner’ (3.31), ‘software for the ergonomic assessment procedure’ (3.12), and ‘excursing regularly’ (3.04). The performance of sub-factors of psychological risk management was in the order of ‘proper job design’ (3.85), ‘unambiguous job roles’ (3.81), ‘organizational time-outs’ (3.54), ‘conducting workshops with managers’ (3.08), and ‘greater latitude’ (2.27) (Figure 5).

### 4.6. The Improvement Assessment of Risk Management

**Figure 6:**
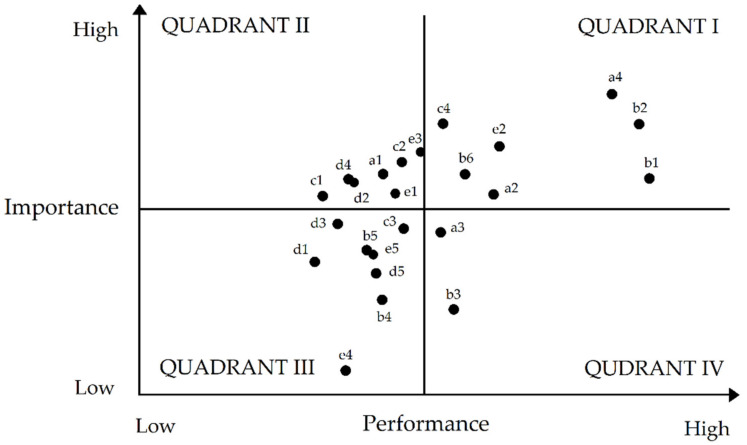
Resultant IPA Matrix.

According to the analysis (Figure 6), the following factors demonstrated a degree of both high importance and high performance: the promotion of human–machine system design; conforming to the safety of state standards; the regular detection of gasoline concentrations; the presence and use of proper ventilation system and equipment maintenance; establishing clear communication lines for emergency preparedness; organizing daily employee learning on relevant safety knowledge; and proper job design. Factors that indicated a high degree of importance but a low level of performance were strengthening the construction of hardware configuration and availability of respirators. Factors that showed both low degrees of importance and low levels of performance were safety glasses and goggles; other protective wear, such as masks, gloves, aprons and boots; medical surveillance; excursing regularly; conducting customers and vehicles flow in a reasonable manner; software for the ergonomic assessment procedure; greater latitude; and conducting workshops with managers. Lastly, factors that displayed a low degree of importance but a high level of performance were personal protective devices (PPDs); regular hand washing; health care for workers about prevision; proper work shifts and schedules; the management of materials scattered in operating spaces; organizational time-outs; and unambiguous job roles (Table 4).

## 5. Discussion

The results of this study reveal that gas station workers are exposed to chemical risk very frequently. Thus, chemical risk management should be of first priority, and plenty of published literature confirms it. Its reasons might be that workers perceive high occurrence probabilities and high risk to life from chemical accidents. Such perception and occurrence of accidents are an outcome of the fact that gas station workers deal with potentially dangerous raw materials [2]. Gasoline is the main source of chemical risks. It is extracted from crude oil and consists of several different kinds of chemical compounds, i.e., toluene, xylene, aromatic hydrocarbons and benzene. Among these compounds, benzene is of prime importance. It has unique physical and chemical properties because of which it can develop vapor pressure by converting it into hazardous gases [2]. It is reported that the mucosal lining of the mouth and eye absorbs benzene efficiently, resulting in this type of occupational accident [70,71]. The exposure level of benzene at gas stations is ≤1 ppm (parts per million). At these levels, benzene has myelotoxic effects, causing lower amounts of platelets and leukocytes (including both lymphocytes as well as granulocytes), myeloid cells (progenitor), and decreased levels of hemoglobin [72,73,74].

Systematically, this compound falls under the category of Group 1 compounds. Chemical compounds of this group are carcinogenic in nature, as reported by the International Agency for Research on Cancer (IARC). Susceptibility to this compound also depends on absorption ability, which varies considerably from person to person. Hence, it is important to observe exposure time to benzene as prolonged contact can be lethal [2]. Mitri et al. [75] found that benzene poisoning was detected in Brazilian gas station workers. Cezar-Vaz et al. [2] found that gas station workers have perceived chemical risks, and they would relate these chemical risks with the occurrence of occupational accidents as dangerous indicators of their work environment. A majority, i.e., 93.7% of these gas station workers, understood that there are serious chemical risks at the gas station. Published literature confirms and reports the same types of risks reported in this study. Rajapakse [76] found that personal exposure to volatile organic compounds at a gas station was much higher, and this had deleterious health effects. Similarly, Zamanian et al. [77] reported that there are harmful outcomes of occupational exposure to petrol, as evaluated by liver function and blood parameters among gas station workers. Silvestre et al. [78] believe there might be health problems for female gas station workers too.

Ebrahemzadih et al. [79] explained that gas station workers are exposed to hazardous vapor, which means that the concentration of air benzene was higher than the permissible standard rate recommended by the National Technical Committee of Occupational Health (0.5 PPM). Santillán [80] described the chemical risk posed by organic vapors of fuel to gas station attendants, causing occupational diseases in the medium or long term. Huibin et al. [81] found that the explosion of dry gas desulfurization tower materials and the fire caused by the leakage of LPG storage tank can cause a maximum injury radius of 199 m, posing a significant threat of chemical risk. Xianlin et al. [82] used the concentration risk rate to characterize the risk degree of toxic and harmful pollutants exceeding environmental standards in the petrochemical industry, and more systematically sorted out the risk assessment and characterization methods of China’s petrochemical industry. Xiaoran et al. [83] used the risk assessment method to evaluate the control effect of occupational hazards on China’s petrochemical projects. The results showed that the risk of occupational hazards related to benzene, toluene, xylene, hydrogen sulfide and ammonia were at a medium risk level. In addition, solvent gasoline and liquefied petroleum gas were also factors likely to cause occupational diseases. Oliveira-Martins and Grisolia [84] discovered toxicity and genotoxicity in wastewater from gasoline stations.

Taking these chemical risks into consideration, various management practices have been suggested by researchers. All these measures aim to reduce chemical exposure by using protective equipment [2,6]. The use of covering gadgets has been suggested to protect workers from benzene exposure [85]. Despite the high possibility of chemical risk at gas stations, practical implementation of preventive management measures is very rare in China. A common observation at filling stations is that workers wear gloves most of the time and only occasionally use other protective equipment. Mostly, workers having low levels of education work at gas stations, and so they do not have a clear idea about occupational health. That’s why we have tried our best to get data for this study from highly qualified workers who have a more solid perception about risk and occupational health.

In order to safeguard the occupational health of gas station workers, physical risk management is the second most important factor to be considered, according to our respondents. The diverse physical risks faced by gas station workers include noise, radiation, vibrations and extreme temperatures. Continuous exposure to these risks results in critical health problems such as tinnitus, dizziness, decreased hearing acuity and even cancer [3,35,39,86,87]. To counter these risks, various tactics have been presented in the published literature. Researchers urge the use of PPD and emphasize on hardware configuration along with the promotion of human–machine system design [47,53,87]. The occurrence of physical risk management in China is rational. This economy has a very huge transportation system consisting of large transportation vehicles. These heavy vehicles generate vibrations of high magnitude. Moreover, mostly gas stations are situated on grand trunk roads, where the mostly bad condition of roads cause additional noise and vibration. Although China is the fastest-growing economy in the world, there is still a lot to improve about it. In terms of priority ranking, this study ranked psychological, biological and physiological risk management as the third, fourth and fifth management factors. Risk factors such as anxiety, irritability, sadness, bacteria, viruses, parasites and fatigue are of central importance in order to protect the occupational health of gas workers [2,4,47]. Various precautionary measures have been suggested in published literature [6,56,58]. However, practical implementation of such measures is yet to become a prerequisite in China.

From the results of IPA’s sub-factor analysis, detection, maintenance and communication during an emergency are the sub-factors of a gas station’s chemical risk management with higher importance and performance, when compared with other sub-factors. The reason might be that prevention is better than cure. Many researchers believe that detection, maintenance and communication during an emergency should be major considerations of a gas station’s chemical risk management [2,88,89,90,91]. The human–machine system and national safety standard are the sub-factors of a gas station’s physical risk management with higher importance and performance, when compared with other sub-factors. The reason might be that automation and standardization should be important physical risk management practices. Bezrodny et al. [92,93] and Kim et al. [94] suggested that the human–machine system and national safety standard should be major considerations of a gas station’s physical risk management. Bezrodny et al. [92,93] elaborated that complex human–machine systems could help the gas station control system structure improvement and control.

Hai [95] proposed that China’s gas stations still have a series of safety problems, such as inadequate implementation of safety management standards, slow investment of safety funds, and lack of employee initiative towards implementing safety standards, which directly or indirectly lead to physical risks for workers. Rongxue et al. [96] explained that centralized and decentralized strategies should be adopted to manage gas station in China. Gas stations in China lack monitoring of passing vehicles, equipment operation and processing of on-site safety parameters such as temperature and liquid level. Therefore, use of automatic emergency responses like turning on the audible and visual alarm device is very necessary. At the same time, the possible disastrous consequences of hazard sources (i.e., death, serious injury and minor injury) are simulated and analyzed, so as to provide decision-making inputs and support for accident emergency rescue.

Safety knowledge training and learning are the sub-factors of a gas station’s biological risk management, with higher importance and performance when compared with other sub-factors. Its reasons might be that training and learning help to form common biological risk management values and action norms. Ramirez-Mindez et al. [97] and Moura-Correa et al. [91] suggested that safety knowledge training and learning should be the major considerations of a gas station’s biological risk management. Youcheng et al. [98] considered that the ability of employees’ operation safety needs to be enhanced when studying the operation safety risk management level of Chinese petroleum enterprises. It is necessary to complete the transformation of employees from “want me to be safe” to “I want to be safe”, and from passive acceptance to active participation through a series of measures such as training grass-roots employees and building a safety culture. Job design is the sub-factor of a gas station’s psychological risk management, with higher importance and performance when compared with other sub-factors. Its reasons might be that job design helps to comprehensively deal with the norms of work content, method, and relations to meet the psychological needs of a gas station’s employees. There are no sub-factors of a gas station’s physiological risk management with most importance and performances, when compared with other sub-factors. Its reasons might be that the gas station’s employees might not care about physiological risk management.

Like most of the other published research, this study does have some potential hitches. For instance, its analytical data were sourced only from the Yangtze River delta region, which limits its ability to generalize results with other fast-developing provinces or regions of China. Thus, there is potential for further research by gathering data on a large scale from more provinces and regions of China. Furthermore, in order to clarify the relationship between occupational risk management and the health of workers, the research needs to evaluate long-term performance of risk management. To achieve this task, other statistical methods with particular focus on long-term management performance can be used in future research. Besides, the statistical techniques used in this study, viz., AHP and IPA, do have some problems. For instance, the scale used in them is ridiculous. It can only be used to judge accurate qualities. Sometimes, the judgements are obscure and cannot be quantified by numerical numbers. Hence, in these conditions, the use of these methods is not authentic. These models only evaluate direct data models, in which information and yield specifically correspond to each other. It cannot disentangle straight models in which information and yield do not directly correspond with each other. Furthermore, it does not cogitate dangers and uncertainties in making choices, but just focuses on stakeholder’s intuition [99]. Besides, these statistical routines do not recognize tradeoffs and their exact relative magnitude [100]. These models’ rank prioritization method is also inadequate [101].

The research outcomes of this study act as a signpost for making managerial strategies. Thus, results have many important as well as practical managerial implications. For instance, this study conducted a performance evaluation of gas station workers’ risk response stratagem and subsequently evaluated improvement priorities. For managers, worker satisfaction is of central importance in any industry. Workers will perform better if their occupational health is secured through better management practices. It will not only lead to the development of the industry but also have a positive impact on the national economy. However, finding appropriate directions for management improvement is not easy. On the other hand, application of chaotic management systems will not bring desirable results. Thus, this study strives to deliver a more efficient system for managing risk by adopting a method of prioritizing and identifying performance points. Accordingly, the main and sub-risk management factors can be targeted to improve the occupational health of gas station workers.

## 6. Conclusions

The core objective of this study was to explore occupational health risks faced by gas station workers. It was found that there are diverse kinds of risk to the health of these workers. On the other hand, risk management practices are scant, and needs to be properly oriented. Thus, it is the need of the hour to take concrete steps to safeguard the occupational health of gas station workers, which can only be achieved through proper intervention of the ruling authorities.

## Figures and Tables

**Table 1:** Characteristics of study subjects.

Category		Frequency	Percent
Marital status	Single	64	30.8
	Married	144	69.2
Gender	Male	144	69.2
	Female	64	30.8
Working Experience	1~3	80	38.5
	Over 3 years	128	61.5
Region	Zhejiang Province	96	46.2
	Shanghai	56	26.9
	Jiangsu Province	56	26.9
Work shifts and schedule	Day time	168	80.8
	Night time	40	19.2
Schooling	Middle School	16	7.70
	High School	56	26.9
	Higher education, incomplete	136	65.4
	Total	208	100.0

**Table 2:** The importance of risk response schemes.

Category	Importance	Ranking
Physical risk management	0.219	2
Chemical risk management	0.341	1
Biological risk management	0.137	4
Physiological risk management	0.116	5
Psychological risk management	0.186	3

**Table 3:** Performance Analysis of Main Factors.

Category	Average	Ranking
Physical risk management	3.19	4
Chemical risk management	3.81	1
Biological risk management	3.65	2
Physiological risk management	3.50	3
Psychological risk management	2.85	5

**Table 4:** IPA Analysis.

Code	Factor	Importance Weights (%)	Performance Weights (%)	Quadrant
a1	Personal protective device (PPD)	0.033	3.654	IV
a2	Promote human–machine system design	0.058	3.500	I
a3	Strength the construction of hardware configuration	0.045	3.231	II
a4	Conform to the safety of state standard	0.084	4.231	I
b1	Regular detection of gasoline concentration	0.092	3.615	I
b2	Proper ventilation system and equipment maintenance	0.090	4.000	I
b3	Availability of respirators	0.048	2.692	II
b4	Safety glasses and goggles	0.031	2.769	III
b5	Other protective gadgets such as masks, gloves, apron and boots	0.030	2.962	III
b6	Establishment of clear communication lines for emergency preparedness	0.051	3.654	I
c1	Washing hands regularly	0.020	3.500	IV
c2	Healthcare for workers about prevision	0.036	3.731	IV
c3	Medical surveillance	0.035	3.269	III
c4	Organize employee daily learning relevant safety knowledge	0.046	4.000	I
d1	Excursing regularly	0.016	3.038	III
d2	Proper work shifts and schedule	0.027	3.577	IV
d3	Conducting customers and vehicles flow reasonably	0.021	3.308	III
d4	Management of materials scattered on operating space	0.025	3.615	IV
d5	Software for the ergonomic assessment procedure	0.028	3.115	III
e1	Organizational time-outs	0.034	3.538	IV
e2	Proper job design	0.059	3.846	I
e3	Unambiguous job roles	0.041	3.808	IV
e4	Greater latitude	0.023	2.269	III
e5	Conducting workshops with managers	0.029	3.077	III

## Data Availability

The data presented in this research are not publicly available due to participant’s privacy.
